# Influence of Perceived Behavioural Control and Knowledge on Nursing Students’ Intention to Prevent Nosocomial Infections: A Cross-Sectional Study

**DOI:** 10.3390/nursrep16040130

**Published:** 2026-04-13

**Authors:** Erwin, Dedi Afandi, Usman M. Tang, Aria Gusti

**Affiliations:** 1 Doctoral Postgraduate Programme in Environmental Science, Universitas Riau, Pekanbaru 28293, Indonesia; 2 Forensic Medicine and Medicolegal Department, Faculty of Medicine, Universitas Riau, Pekanbaru 28293, Indonesia; dediafandi4n6@gmail.com; 3 Department of Aquaculture, Faculty of Fisheries and Marine, Universitas Riau, Pekanbaru 28293, Indonesia; usman.mtang@lecturer.unri.ac.id; 4 Department of Public Health, Universitas Andalas, Padang 25163, Indonesia; ariagusti@ph.unand.ac.id

**Keywords:** nosocomial infections, nursing students, perceived behavioural control, knowledge, personal protective equipment, intention

## Abstract

**Background:** Hospital-acquired infections (HAIs) pose significant safety risks, making nursing students’ behavioural intention during clinical rotations vital for prevention. **Objective:** To analyze the influence of Perceived Behavioural Control (PBC) and knowledge on students’ intention to maintain a safe clinical environment. **Methods:** A cross-sectional design was conducted with 242 nursing students at a Type A referral hospital in Pekanbaru, Indonesia. Participants were selected via simple random sampling. Data were collected using validated questionnaires measuring PBC (six indicators), knowledge (three subscales), and behavioural intention. Statistical analysis involved Chi-square tests for unadjusted Odds Ratios (OR) and binary logistic regression to calculate adjusted Odds Ratios (AOR) by entering all variables into the model simultaneously. **Results:** The majority of participants demonstrated high intention (66.5%) and high PBC (83.9%). In the univariate analysis, all six PBC indicators and general nosocomial knowledge were significantly associated with high intention (*p* < 0.05), with staff direction (OR = 5.96) and specific training (OR = 4.94) showing the strongest independent effects. However, when all environmental and cognitive variables were entered into the regression model simultaneously, only knowledge of personal protective equipment (PPE) use remained a significant independent factor (AOR = 2.66; 95% CI: 1.40–5.06, *p* = 0.003). The unadjusted OR emphasized the isolated influence of each factor, whereas the adjusted OR showed that technical knowledge was the only variable to retain significance after controlling for other factors. **Conclusions:** Technical knowledge regarding PPE use is the primary independent driver of nursing students’ intention to maintain a safe clinical environment. While environmental support and general knowledge are important foundational elements, clinical education should prioritize practical, technical training in protective measures to translate knowledge into behavioural intention effectively.

## 1. Introduction

Hospital-acquired infections (HAIs) are highly prevalent worldwide, with point estimates up to 15.5% in developing countries and intensive care unit rates exceeding 30%, leading to prolonged stays, higher mortality, and substantial economic burden for health systems [1,2]. For individual hospitals, HAIs can add 7–8 extra inpatient days per case and thousands of dollars in direct costs, especially for pneumonia, bloodstream, and surgical-site infections. At the same time, national estimates in high-income settings reach billions annually [3,4]. In clinical settings, nursing students represent a large portion of the healthcare workforce in training, often providing direct bedside care during their rotations [5,6]. Because students are still developing their clinical competencies, their adherence to infection control protocols is vital. However, maintaining a safe clinical environment is a complex task that depends not only on a student’s willingness but also on the interaction between their technical understanding and the clinical environment itself [7,8].

While various theoretical frameworks, such as the Social Cognitive Theory or the Health Belief Model, are frequently employed in medical education research to explain behavioural changes [9], this study specifically prioritizes Perceived Behavioural Control (PBC) within the Theory of Planned Behaviour (TPB) framework [10]. This choice is justified because PBC uniquely captures the dual influence of internal self-efficacy and external situational constraints—such as the availability of facilities and clinical supervision which are critical determinants for nursing students navigating high-pressure clinical rotations [11,12]. Unlike models that focus solely on individual beliefs, PBC accounts for the practical ‘ease or difficulty’ of performing a behavior in a real-world hospital setting where students have limited autonomy. This makes it a more robust predictor for infection control intentions in environments where resource availability and staff direction directly shape a student’s capacity to act.

The theoretical foundation of this study is the TPB, which was originally developed by Icek Ajzen as an extension of the Theory of Reasoned Action (TRA) [10,13]. TPB is a generalized behavioural model designed to predict human behaviour across various domains by identifying three core determinants of behavioural intention: attitude toward the behaviour, subjective norms, and PBC [10]. In the context of clinical education, PBC is a critical construct because it refers to an individual’s perception of the ease or difficulty of performing a specific behavior based on their internal capabilities and external resources. For nursing students, behavioural intention serves as a primary predictor of actual clinical safety practices. While TPB is a general model, extensive research in infection control and patient safety has consistently demonstrated that PBC is a strong predictor of safety-related intentions, such as hand hygiene adherence, adverse event reporting, and the use of personal protective equipment (PPE). For nursing students on clinical rotation, PBC is significantly shaped by external environmental factors and institutional support [14,15]. These factors include the availability of facilities (e.g., hand-hygiene stations), the clarity of directions provided by the Infection Prevention and Control (IPC) team, and the level of supervision and motivation from senior clinical staff [14,16]. By focusing on PBC, this study examines how the interaction between students’ technical understanding and their clinical environment influences their intention to maintain a safe environment.

In health professions education, competence is commonly conceptualized using the Knowledge–Skills–Attitudes (KSA) framework, which distinguishes cognitive knowledge, procedural skills, and professional attitudes as complementary domains of learning [17]. Within this framework, infection prevention and control competencies involve knowledge of infection mechanisms and prevention principles, practical skills such as the correct use of PPE, and attitudes reflecting commitment to patient safety and adherence to infection-control standards [18,19]. It encompasses a general theoretical understanding of nosocomial infections, awareness of infection transmission mechanisms, and technical proficiency in the use of PPE [20]. Students often show “moderate” or “fair” overall knowledge, but with notable gaps in definitions of HAIs, timing of onset, and chain of infection [21,22]. In the present study, the knowledge construct refers specifically to the cognitive domain, focusing on students’ conceptual understanding of healthcare-associated infections (HAIs) and infection prevention practices. This includes general knowledge of nosocomial infections, knowledge of infection transmission, and knowledge of appropriate PPE use. The knowledge variable therefore reflects theoretical understanding of infection-prevention principles rather than the practical performance of infection-control procedures.

Despite the critical role of these factors, there is limited evidence on how specific sub-dimensions of knowledge and environmental control independently influence behavioural intention among nursing students, particularly in high-complexity settings. Most existing research focuses on general knowledge rather than the specific technical skills that drive commitment to safety [23,24,25]. Most studies treat knowledge as a single IPC or disease-specific score, rather than separating it into theoretical vs. technical skill domains [24,26]. Higher overall IPC or standard precaution knowledge is associated with better compliance or intentions in some studies [23,26]. Nursing students with higher knowledge scores demonstrated better performance in IPC practices, indicating a direct relationship between knowledge and preventive behaviours [27,28]. However, the effect is inconsistent and sometimes even negative for high-risk infectious disease care [26].

Based on the theoretical framework and previous literature, the following hypotheses were proposed. First, (H1) perceived behavioural control indicators (availability of facilities, staff direction, supervision, training, and institutional motivation) positively influence nursing students’ intention to maintain a safe clinical environment. (H2). Knowledge of infection prevention positively influences nursing students’ intention to maintain a safe clinical environment, and (H3) Technical knowledge related to PPE use exerts a stronger influence on behavioural intention compared with other knowledge domains. Therefore, this study aims to analyze the influence of Perceived Behavioural Control (PBC) and knowledge on students’ intention to maintain a safe clinical environment. Identifying these specific drivers is essential for refining clinical education and hospital induction programmes to ensure the effective prevention of hospital-acquired infections.

## 2. Materials and Methods

### 2.1. Study Design

The study used a cross-sectional approach conducted from February to October 2023. The participants were nursing students at nursing education institutions in Pekanbaru, Indonesia, who were in their professional practice stage. In Indonesia, this stage refers to the Ners professional programme, which represents the final clinical training phase required for nursing licensure. Prior to entering this programme, students complete approximately four years of undergraduate academic education (Bachelor of Nursing), which focuses on foundational nursing theory, basic clinical competencies, and laboratory-based training. After completing the academic stage, graduates undertake the Ners professional programme, which typically lasts around one year and consists of supervised clinical rotations across multiple hospital departments. Upon completion of this professional training, graduates become eligible to take the national nursing competency examination and apply for professional licensure as registered nurses. The primary research site was Arifin Achmad Hospital, selected because it serves as the main teaching hospital and Type A referral facility in Riau Province, offering students exposure to complex medical cases and a structured environment for clinical application.

### 2.2. Population and Sample

The study population consisted of approximately 420 nursing students enrolled in a nurse profession programme. From this population, 242 nursing students undergoing clinical rotations were recruited via simple random sampling. All students who were actively participating in clinical practice were eligible, while those on academic leave were excluded.

The required sample size was determined using G*Power version 3.1.9.7 with a logistic regression approach. The calculation assumed a medium effect size (Cohen’s f^2^ = 0.15, equivalent to an odds ratio of approximately 1.5), a significance level of 0.05, a statistical power of 0.80, and two predictors (perceived behavioural control and knowledge). Based on these parameters, the minimum required sample size was 107 participants. To increase statistical power and account for potential non-response, the study recruited the entire accessible population of 242 nursing students, exceeding the minimum required sample size and ensuring robust analysis.

### 2.3. Research Instrument

The questionnaire began with a demographic survey to capture participants’ background characteristics. This section included items on age, gender, length of clinical rotation, programme type (regular or extension), ethnicity, and religion. These variables were collected to provide descriptive information about the study population and to allow for subgroup analysis.

The main instrument consisted of three sections: Intention, Perceived Behavioural Control (PBC), and Knowledge (Table 1). The questionnaire was developed based on a literature review and previous research instruments, was validated by a panel of experts, and was pilot-tested among nursing students to ensure validity and reliability. The internal consistency of the instrument and each sub-dimension was estimated using Cronbach’s alpha coefficients. All instrument items can be seen in the Supplementary Files.

1. Intention Scale

The indicators for the intention variable were adapted from questionnaires developed by Kumar (2012) [29], Gusti (2016) [30], Alrubaiee (2021) [31], the Indonesian Ministry of Health Regulation [32,33] and Livshiz-Riven, Hurvitz, and Ziv-Baran (2022) [34], all of which focus on behaviours related to nosocomial infection control. In this study, intention refers to the willingness to perform four key indicators of safe environmental management behaviour (safety environment behaviour) in preventing nosocomial infections, namely: hand hygiene, proper segregation of infectious and non-infectious waste, appropriate use of PPE, and the provision of education. The initial list of questionnaire items was discussed with experts. Based on their feedback and recommendations, the items were revised and refined. The final version of the questionnaire was then subjected to validity and reliability testing.

The intention scale comprised 21 items, adapted from Gusti and colleagues’ work based on Ajzen’s theory [10,30]. Each item used a 5-point Likert scale, ranging from 1 = strongly disagree to 5 = strongly agree, with scores adjusted according to whether the construct was positive or negative. Higher scores indicated stronger behavioural intention, and the results were categorized into low and high levels. In this study, the instrument demonstrated good validity, with Pearson r ranging from 0.334 to 0.774 (r = 0.138 for the 242-sample table). Furthermore, the Cronbach’s alpha result was 0.873.

2. Perceived Behavioural Control (PBC) Scale

The development of the PBC instrument was conducted using both quantitative and qualitative approaches. The quantitative approach employed an elicitation method, which involved distributing questionnaires and conducting direct interviews to explore students’ perceived behavioural control regarding their intention to perform safe environmental management behaviours (safety environment behaviour) in preventing nosocomial infections.

The elicitation items were developed based on a literature review [10,30,35,36]. Two main open-ended questions were posed to respondents: (1) “What factors make (or would make) it easier for you to implement safe environmental management behaviours in preventing nosocomial infections?” and (2) “What difficulties do you face in implementing safe environmental management behaviours in preventing nosocomial infections?” The elicitation process was conducted with 30 students. Based on the results, the ten most frequently mentioned responses were identified. These findings were then reviewed and discussed with experts. The outcomes of the expert review were used to develop the final instrument, which was subsequently tested for validity and reliability.

The PBC scale included 6 items developed from an elicitation study that measured students’ perceptions of ease or difficulty in performing safe environmental behaviours. Each item used a 5-point Likert scale (1 = strongly disagree to 5 = strongly agree). Indicators covered adequacy of facilities, directions from infection prevention and control staff, supervision, training, and motivation. The mean score was categorized into low and high PBC. In this study, the instrument demonstrated good validity, with Pearson r ranging from 0.580 to 0.819 (r = 0.138 for the 242-sample table). Furthermore, the Cronbach’s alpha result was 0.894.

3. Knowledge Scale

The questionnaire assessing knowledge of safe environmental management (safety environment behaviour) in preventing nosocomial infections consists of three manifest variables: (1) knowledge of nosocomial infections, (2) knowledge of infection transmission, and (3) knowledge of the use of PPE in preventing nosocomial infections. The initial construct of this scale was developed based on the Guidelines for the Management and Prevention of Nosocomial Infections published by Arifin Ahmad hospital [32,33].

Based on the key points outlined in the guideline, the author conducted an elicitation process by compiling a list of questionnaire items. This initial set of items was then subjected to expert review, involving the Infection Prevention and Control (PPI) team of Arifin Ahmad Hospital, one doctoral expert in medical-surgical nursing, one doctoral expert in nosocomial infections, and two nursing practitioners. Based on the experts’ feedback, revisions were made to improve the questionnaire items. The final version of the instrument was subsequently pilot-tested to assess its validity and reliability.

The knowledge scale consisted of 15 items divided into three sub-dimensions: general knowledge of nosocomial infections, knowledge of infection transfer, and knowledge of personal protective equipment use. Each item was scored on an interval scale, with a total score ranging from 0 to 15. Results were categorized into low and high levels based on the mean score. In this study, the instrument demonstrated good validity, with Pearson r ranging from 0.235 to 0.569 (r = 0.138 for the 242-sample table). Furthermore, the Cronbach’s alpha result was 0.7.

### 2.4. Data Collection

Ten faculty members from the nursing school were selected to assist with the distribution of the survey. These individuals were senior lecturers and clinical instructors who maintained close contact with the researchers and served as supervisors during student clinical rotations. Participation was voluntary and anonymous, and students were informed that their responses would not influence their academic evaluation or clinical assessment to minimize potential social desirability bias.

The investigators converted the questionnaire into a secure online format using Google Forms, enabling easy distribution and monitoring. All items were required to be completed, and the system prevented multiple submissions from the same account to ensure data integrity. Prior to the formal survey, training was provided to these faculty members to familiarize them with the questionnaire and the recruitment procedure. Each faculty member was asked to recruit at least 20 nursing students during the same period. The survey link was distributed via WhatsApp and institutional email, which are widely used communication platforms among nursing students in Indonesia. Faculty members shared the survey link directly with student groups during clinical rotation briefings. Data collection was monitored in real time through the Google Forms management dashboard. A preliminary survey was conducted among 20 students to estimate the average completion time, which was approximately six minutes, with the shortest time exceeding four minutes. To ensure response quality, questionnaires completed in less than three minutes or with more than two-thirds of items missing were excluded. During the data screening process, one questionnaire was excluded because the completion time was less than three minutes, which did not meet the predefined quality criteria. No responses had more than two-thirds of items missing. Therefore, a total of 242 valid questionnaires were retained for the final analysis.

### 2.5. Data Analysis

The statistical analysis was conducted using IBM SPSS version 27.0. Descriptive statistics were used to summarize demographic characteristics and variable distributions. Analysis of the relationship between variables was carried out using the Spearman rank test because the data were ordinal and not normally distributed. In addition, a multivariate analysis was performed using binary logistic regression to examine the influence of perceived behavioural control and knowledge on students’ intention to engage in safe environmental behaviours. A significance level of *p* < 0.05 was applied. The Hosmer–Lemeshow goodness-of-fit test was used to assess model adequacy; values greater than 0.05 indicated acceptable fit.

For analytical purposes, composite scores for each construct were calculated by averaging the responses across items within the same scale. To facilitate interpretation and to enable the use of binary logistic regression, the resulting scores were subsequently categorized into two groups (“low” and “high”) using the mean value as the cut-off point. This categorization approach has been commonly applied in health and behavioural research to simplify the presentation and interpretation of odds ratios, particularly in applied settings where distinguishing between relatively lower and higher levels of constructs is of practical interest [37,38,39,40]. In addition, this methodological choice is consistent with prior applications of the Theory of Planned Behaviour (TPB) in health professions education, where dichotomization has been used to facilitate clearer interpretation of behavioural intention outcomes. For example, Harris (2021) [41] provides a primer on binary logistic regression in medical education research, highlighting its utility in classifying behavioural outcomes into binary categories. Similarly, Twisk (2025) [42] emphasizes that logistic regression is inherently designed for dichotomous outcomes, and dichotomization can be appropriate when the research objective is to contrast groups with “high” versus “low” levels of intention or control. However, we acknowledge that dichotomization of continuous or ordinal variables may lead to a loss of information and a potential reduction in statistical power. Therefore, this approach should be interpreted with caution, and future studies are encouraged to retain the full scale of measurement or consider more advanced analytical techniques.

### 2.6. Ethical Consideration

This study was reviewed and approved by the Ethics Committee of the Faculty of Nursing, Universitas Riau, with approval number 296.C/UN19.5.1.8/KEPKFKp/2023. The research was conducted in full compliance with the ethical principles of the Declaration of Helsinki and relevant national and institutional guidelines. Participation in the study was entirely voluntary, and written informed consent was obtained from all respondents prior to data collection.

Participants were provided with clear and comprehensive information regarding the objectives, procedures, potential risks, and benefits of the study before agreeing to participate. They were assured that all data collected would be treated with strict confidentiality and anonymity. Personal identifiers were removed, and the data were securely stored and accessed only by the research team for academic purposes. Furthermore, participants were informed of their right to refuse to participate or to withdraw from the study at any stage, without academic, personal, or professional consequences. No form of coercion or undue influence was applied during recruitment or data collection. The study posed minimal risk to participants, and all efforts were made to protect their dignity, rights, and well-being throughout the research process.

## 3. Results

### 3.1. Demographic Characteristics

Out of the total population of approximately 420 nursing students, 243 questionnaires were initially collected. During the data screening process, one questionnaire was excluded because the completion time was less than three minutes, which did not meet the predefined quality criteria. All remaining questionnaires met the screening criteria, with no responses containing more than two-thirds missing data. Therefore, a total of 242 valid responses (57.6%) were included in the final analysis.

Table 2 presents the characteristics of the respondents in relation to age, gender, programme type, clinical rotation length, ethnicity, and religion. The majority of the respondents were female (83.1%) with a mean age of 28.48 years (SD = 7.37). Regarding clinical experience, most students had rotations lasting 6 months or longer (94.6%). The distribution between programme types was nearly equal, with 122 students (50.4%) enrolled in the regular course and 120 students (49.6%) in the extension course. Ethnically, the respondents were dominated by Melayu students (33.1%), followed by Minang (31.0%), Java (15.3%), Batak (14.9%), and others (5.7%). In terms of religion, the majority identified as Muslim (87.2%), while 12.8% were Christian or Catholic. None of the demographic variables showed a statistically significant association with behavioural intention (*p* > 0.05).

### 3.2. Description and Association Between PBC Indicators and the Level of Knowledge with Intention

A total of 203 (83.9%) students reported high overall PBC, with the most prevalent indicator being the availability of adequate facilities (93.0%), as shown in Table 3. In contrast, high overall knowledge was found in 136 (56.2%) participants. Knowledge levels were notably low for general nosocomial concepts (78.9%) and infection transfer (64.9%), while knowledge of PPE at 57.0%. Overall, 161 (66.5%) students demonstrated high intention to maintain a safe environment.

The bivariate analysis showed that all PBC indicators were significantly associated with intention (*p* < 0.05). Students who perceived high staff directions (*p* = 0.001) and specific training (*p* = 0.002) exhibited higher behavioural intention. Among the knowledge subscales, only PPE use was significantly associated with intention (*p* < 0.001), with students with high technical knowledge more likely to have high intention (34.7%) than those with low knowledge (8.3%). General nosocomial knowledge and knowledge of infection transmission did not show statistically significant associations with intention (*p* = 0.063 and *p* = 0.090, respectively).

### 3.3. Influence of PBC Indicators and Level of Knowledge on Intention

In the univariate analysis, all six PBC indicators and general nosocomial knowledge were significantly associated with behavioural intention (*p* < 0.05), with staff directions (PBC 3) and specific training (PBC 4) showing the strongest influence (OR = 5.96 and 4.94, respectively). The “general nosocomial knowledge” subscale included items assessing students’ understanding of the definition of nosocomial infections, standard precautions, hand hygiene as a pillar of infection prevention, and basic infection-related complications such as phlebitis. However, the multivariate analysis revealed that only knowledge of PPE use (Sub 3) remained a significant independent factor (*p* = 0.003). After PBC indicators and knowledge subscale were entered into the regression model simultaneously, students with high technical knowledge of PPE were 2.66 times more likely to demonstrate high intention to maintain a safe clinical environment (AOR = 2.66; 95% CI: 1.40–5.06). In contrast, the remaining PBC indicators and other knowledge subscales did not exert a statistically significant independent influence in the final adjusted model (*p* > 0.05) (Table 4).

## 4. Discussion

The findings of this study provide partial support for the proposed hypotheses and offer important insights into the determinants of nursing students’ intention to maintain a safe clinical environment. Overall, the results indicated that several environmental and cognitive factors were associated with behavioural intention in the univariate analysis, including all six indicators of PBC and general knowledge of nosocomial infections. However, these associations did not persist in the adjusted regression model.

The first hypothesis, which predicted that higher perceived behavioural control would be associated with stronger behavioural intention, was supported in the univariate analysis but not in the multivariate model. After controlling for all variables simultaneously, none of the PBC indicators remained statistically significant. This suggests that although environmental supports may initially influence students’ behavioural intentions, their independent effect may diminish when other determinants are considered. The second hypothesis regarding the influence of knowledge on behavioural intention received partial support. Among the three knowledge sub-dimensions examined, only knowledge related to the use of PPE remained a significant independent predictor in the multivariate analysis. This finding indicates that not all domains of infection-prevention knowledge contribute equally to behavioural intention. Then, the third hypothesis, which proposed that different knowledge domains would exert varying effects on intention, was supported. These results highlight the particular importance of technical knowledge related to PPE use in shaping nursing students’ intention to maintain a safe clinical environment.

Compared with previous studies applying the Theory of Planned Behaviour among healthcare students and nurses, the present study provides several novel contributions. Earlier studies have generally focused on the overall relationship between knowledge, attitudes, subjective norms, PBC, and intention. For example, Al Maskari et al. [26] reported that knowledge was correlated with attitudes and perceived behavioural control but showed a negative association with intention, while Sin et al. [14] and Zhong et al. [43] found that subjective norms and perceived behavioural control were the dominant predictors of behavioural intention in infection-control behaviours. Similarly, Kim et al. [36] demonstrated that TPB constructs such as attitude, subjective norms, and perceived behavioural control were positively associated with intention and infection-control performance. In contrast, the present study extends this literature by analyzing knowledge as a multidimensional construct and examining specific environmental indicators of perceived behavioural control among nursing students during clinical rotations.

In this study, staff directions and specific training emerged as the strongest influences, suggesting that institutional guidance and structured learning opportunities play an important role in shaping students’ initial behavioural intentions (in univariate analysis, Table 4). However, in the multivariate analysis, only knowledge of PPE remained a significant independent predictor, with students who demonstrated high technical knowledge being 2.66 times more likely to report a strong intention to engage in safe environmental practices. This finding indicates that while external support factors such as supervision and training may encourage intention at the surface level, it is the internalized and practical knowledge of PPE use that ultimately sustains behavioural commitment. The result aligns with Ajzen’s theory of planned behaviour [10], which emphasizes that intention is most strongly influenced by specific, actionable knowledge and perceived control over behaviour.

When examining factors associated with behavioural intention to maintain a safe clinical environment, knowledge of PPE use was significantly associated with stronger intention. This finding is consistent with studies conducted in Malaysia, Taiwan, and Turkey, which found that nursing students with higher technical competence in infection control procedures were more likely to adhere to safe practices in clinical settings [44,45,46]. This stronger intention may arise from students’ familiarity with the practical use of PPE and its direct relevance to their daily clinical tasks [34]. Interestingly, while staff directions and specific training initially showed strong associations in the univariate analysis, they did not remain significant in the adjusted model. This suggests that external guidance alone may not be sufficient to sustain behavioural intention unless accompanied by internalized technical knowledge. Understanding this more clearly will require future studies that explore how experiential learning, supervision quality, and students’ intrinsic motivation interact with technical knowledge to shape long-term behavioural outcomes [24,47]. In addition, other contextual factors, such as institutional culture or peer influence, should be considered in future analyses.

Environmental supports likely shape PPE intention indirectly for nursing students on clinical rotation. Among nursing students, PPE-related teaching, simulation, and supervision strengthen technical understanding and practical skills; these are key precursors to safe behaviour rather than stand-alone predictors of intention [48]. Broader evidence from nurses in hospital settings shows that safety leadership and empowering leadership increase safety knowledge and safety motivation, which, in turn, mediate the effect of leadership on safety compliance and participation [49]. Qualitative work with health-care personnel during COVID-19 further indicates that environmental context (PPE availability, workflow design, reminders), leadership commitment, and clear professional roles enhance perceived competence and confidence in PPE use, rather than directly forcing compliance [50,51,52]. These findings should also be interpreted in the context of resource availability. In clinical settings where infection-control resources such as hand hygiene facilities or PPE are limited, perceived behavioural control may be lower, which could potentially weaken students’ behavioural intentions. Therefore, the influence of environmental factors observed in this study may differ in resource-constrained healthcare facilities.

Although PBC has often been reported as a strong predictor of behavioural intention in the Theory of Planned Behaviour (TPB), the present study found that PBC indicators did not remain statistically significant in the adjusted regression model. This discrepancy may reflect the contextual nature of TPB constructs, where the relative contribution of attitude, subjective norms, and PBC can vary depending on the behaviour and the population being studied. Previous research has shown that different TPB components may dominate behavioural intention in different contexts. For instance, a multisite study among junior nurses and final-year nursing students found that subjective norms were the strongest predictor of intention to care for COVID-19 patients, while PBC contributed a smaller proportion of explained variance [43]. Similarly, studies examining infection-prevention behaviours such as hand hygiene have demonstrated that the predictive strength of TPB constructs is not constant across settings, with certain variables becoming less influential when other determinants exert stronger effects [14]. In the context of the present study, the environmental factors represented by PBC indicators, such as supervision, institutional support, and training, may function more as enabling conditions rather than direct determinants of intention. When analyzed together with cognitive variables, particularly technical knowledge related to PPE use, their independent contribution may diminish due to overlapping explanatory mechanisms. This finding suggests that while supportive clinical environments remain important for facilitating infection-prevention practices, students internalized technical competence may represent a more proximal determinant of their intention to maintain a safe clinical environment.

The duration of clinical rotation was also examined as a potential factor influencing behavioural intention (Table 2). However, no statistically significant association was observed between rotation length and intention to maintain a safe clinical environment (*p* = 0.607). This finding should be interpreted cautiously, as the majority of participants had clinical rotations of six months or longer (94.6%), while only a small proportion had shorter rotations (<6 months, 5.4%). Such an uneven distribution may have limited the statistical power to detect meaningful differences between groups. Nevertheless, longer clinical exposure may theoretically contribute to greater familiarity with infection-prevention practices and the clinical workflow [53,54,55,56]. In the present study, however, behavioural intention appears to be more strongly influenced by cognitive factors, particularly technical knowledge related to PPE use, rather than by the duration of clinical rotation itself.

Reviews and empirical studies also highlight that education, regular training, and supervision function as upstream conditions: they improve knowledge and embed PPE within a safety-oriented culture, which then encourages consistent use in day-to-day patient care [52,57,58]. For nursing students rotating through clinical areas, this implies that ward-level supports (preceptor behaviour, role-modelling, debriefing about infection risks, easy PPE access, and visible safety norms) most plausibly influence intention via what students learn and how they perceive the unit’s safety climate, not merely via instructions [59]. The loss of significance for staff directions or institutional encouragement in adjusted models is therefore compatible with partial mediation: environmental supports appear to exert much of their impact by enabling students to acquire and apply PPE knowledge within a supportive clinical safety culture, which then translates into stronger intentions to maintain a safe environment [60].

Despite these important findings, several limitations should be acknowledged. First, the study was conducted in a single nursing institution, which may limit the generalizability of the results to other settings or populations. Second, the cross-sectional design restricts the ability to establish causal relationships between perceived behavioural control, knowledge, and behavioural intention. Third, the use of self-reported data may be subject to social desirability bias, as participants may overestimate their intention to maintain a safe clinical environment.

Finally, the categorization of continuous or ordinal variables into dichotomous groups (“low” and “high”) may resulted in a loss of information and a potential reduction in statistical power. Although this approach was adopted to facilitate interpretation of the results, particularly in the context of logistic regression and odds ratios, it may have limited the sensitivity of the analysis in detecting more nuanced relationships between variables. Therefore, the findings should be interpreted with caution, and future studies are encouraged to retain continuous measurements or apply more advanced analytical approaches to better capture the complexity of behavioural constructs.

## 5. Conclusions

We used a quantitative study to examine the influence of perceived behavioural control and knowledge on nursing students’ intention to maintain a safe clinical environment in Indonesia. The findings indicate that technical knowledge, particularly regarding the correct use of personal protective equipment (PPE), plays an important role in shaping nursing students’ behavioural intentions in infection prevention practices. In addition, the findings highlight that staff direction and supervision during clinical practice are essential components that reinforce students’ ability and confidence to apply PPE appropriately in real clinical situations. While environmental support and general knowledge remain important foundational elements, the combination of practical technical competence and clear guidance from clinical staff is crucial to translating knowledge into consistent behavioural intention. Nursing educators therefore need to build a strong infection prevention learning environment and promote opportunities for theoretical and practical integration through simulation and supervised clinical practice. Strengthening collaboration between educators and clinical staff in guiding, monitoring, and reinforcing PPE use during clinical placements can further enhance students’ adherence to infection prevention measures. Strengthening institutional reinforcement and feedback mechanisms is an effective strategy to improve students’ infection prevention behaviours, enabling them to experience a safer clinical environment and meaningful learning in a way that builds their professional competencies.

## Figures and Tables

**Table 1:** Categorization of Knowledge, Perceived Behavioural Control (PBC), and Intention, Validity, and Reliability.

Variable	Subscale/Indicator	Score Range	Categorization
Intention	Behavioural intention to perform safe environmental practices (21 items)	1–5 Likert	Low/High
Perceived Behavioural Control	Adequate facilities in each patient room (sink, soap for hand hygiene)	1–5 Likert	Low (≤mean)/ High (>mean)
	Directions from the hospital IPC team	1–5 Likert	Low/High
	Directions from the head of the room or structural staff	1–5 Likert	Low/High
	Specific training on managing nosocomial infections	1–5 Likert	Low/High
	Supervision (suboptimal)	1–5 Likert	Low/High
	Motivation from the hospital (minimal)	1–5 Likert	Low/High
Knowledge	General knowledge of nosocomial infections (items 1, 2, 3, 14, 15)	0–5	Low (≤median)/ High (>median)
	Knowledge of infection transfer (items 4, 5, 8, 13, 16)	0–5	Low/High
	Knowledge of personal protective equipment use (items 7, 9, 10, 11, 12)	0–5	Low/High

**Table 2:** Characteristics of the nursing students (*n* = 242).

Variable	Low Intention *n* (%)	High Intention *n* (%)	Total *n* (%)	*p*-Value
Gender				
Female	64 (26.5%)	137 (56.6%)	201 (83.1%)	0.313
Male	17 (7.0%)	24 (9.9%)	41 (16.9%)	
Length of Clinical Rotation				
<6 months	3 (1.2%)	10 (4.2%)	13 (5.4%)	0.607
≥6 months	78 (32.2%)	151 (62.4%)	229 (94.6%)	
Nurse Profession Program				
Regular Course	34 (14.0%)	88 (36.4%)	122 (50.4%)	0.084
Extension Course	47 (19.4%)	73 (30.2%)	120 (49.6%)	
Ethnicity				
Minang	23 (9.5%)	52 (21.5%)	75 (31.0%)	0.370
Batak	13 (5.4%)	23 (9.5%)	36 (14.9%)	
Melayu	32 (13.1%)	48 (19.8%)	80 (33.1%)	
Java	8 (3.3%)	29 (12.0%)	37 (15.3%)	
Others	5 (2.1%)	9 (3.6%)	14 (5.7%)	
Religion				
Muslim	69 (28.5%)	142 (58.7%)	211 (87.2%)	0.647
Christian/Catholic	12 (5.0%)	19 (7.8%)	31 (12.8%)	

Note: Age (Mean, SD) = 28.48 (7.37). All percentages are calculated based on the total sample size (*n* = 242).

**Table 3:** The association between each PBC indicator and the knowledge subscale with intention (*n* = 242).

Variable	Low Intention *n* (%)	High Intention *n* (%)	Total *n* (%)	*p*-Value
PBC Indicators				
PBC 1: Adequate facilities				
Low	11 (4.5%)	6 (2.5%)	17 (7.0%)	0.010 *
High	70 (28.9%)	155 (64.1%)	225 (93.0%)	
PBC 2: IPC team directions				
Low	17 (7.0%)	10 (4.2%)	27 (11.2%)	0.001 *
High	64 (26.4%)	151 (62.4%)	215 (88.8%)	
PBC 3: Staff directions				
Low	13 (5.3%)	5 (2.1%)	18 (7.4%)	0.001 *
High	68 (28.1%)	156 (64.5%)	224 (92.6%)	
PBC 4: Specific training				
Low	13 (5.4%)	6 (2.5%)	19 (7.9%)	0.002 *
High	68 (28.1%)	155 (64.0%)	223 (92.1%)	
PBC 5: Level of supervision				
Low	11 (4.5%)	7 (2.9%)	18 (7.4%)	0.020 *
High	70 (28.9%)	154 (63.7%)	224 (92.6%)	
PBC 6: Hospital motivation				
Low	12 (5.0%)	6 (2.4%)	18 (7.4%)	0.005 *
High	69 (28.6%)	155 (64.0%)	224 (92.6%)	
Knowledge Subscales				
Sub 1: General nosocomial				
Low	70 (28.9%)	121 (50.0%)	191 (78.9%)	0.063
High	11 (4.6%)	40 (16.5%)	51 (21.1%)	
Sub 2: Infection transfer				
Low	59 (24.4%)	98 (40.5%)	157 (64.9%)	0.090
High	22 (9.1%)	63 (26.0%)	85 (35.1%)	
Sub 3: Use of PPE				
Low	61 (25.2%)	77 (31.8%)	138 (57.0%)	<0.001 *
High	20 (8.3%)	84 (34.7%)	104 (43.0%)	

Notes: Abbreviation: PBC, perceived behavioural control; IPC, interprofessional collaboration; PPE, personal protective equipment. All percentages are calculated based on the total sample size (*n* = 242); * significance variable *p* < 0.05.

**Table 4:** Influence of PBC and level of knowledge subscale on intention (*n* = 242).

Variable	Unadjusted OR	95% CI	*p*-Value	Adjusted OR (AOR)	95% CI	*p*-Value
PBC Indicators						
PBC 1: Adequate facilities	4.06	(1.44; 11.42)	0.008	0.41	(0.03; 5.94)	0.513
PBC 2: IPC team directions	4.01	(1.74; 9.24)	0.001	2.26	(0.66; 7.71)	0.192
PBC 3: Staff directions	5.96	(2.05; 17.39)	0.001	3.64	(0.54; 24.51)	0.185
PBC 4: Specific training	4.94	(1.80; 13.54)	0.002	4.32	(0.28; 65.68)	0.293
PBC 5: Level of supervision	3.46	(1.29; 9.29)	0.014	0.23	(0.02; 3.40)	0.285
PBC 6: Hospital motivation	4.49	(1.62; 12.46)	0.004	2.02	(0.34; 12.19)	0.442
Knowledge Subscales						
Sub 1: General nosocomial	2.10	(1.01; 4.36)	0.046	1.65	(0.73; 3.71)	0.228
Sub 2: Infection transfer	1.72	(0.96; 3.09)	0.067	1.38	(0.72; 2.65)	0.338
Sub 3: Use of PPE	3.33	(1.84; 6.02)	<0.001	2.66	(1.40; 5.06)	**0.003**

**Note**: Reference group is “Low” (0). Statistically significant values (*p* < 0.05) are in bold. Abbreviation: PBC, perceived behavioural control; IPC, interprofessional collaboration; PPE, personal protective equipment.

## Data Availability

The data supporting the findings of this study are not publicly available due to ethical considerations and participant privacy protection.

## Supplementary Materials

The following supporting information can be downloaded at: https://www.mdpi.com/article/10.3390/nursrep16040130/s1, Table S1: All Instruments used.

## Public Involvement Statement

No public involvement in any aspect of this research.

## Guidelines and Standards Statement

This manuscript was drafted in accordance with the Strengthening the Reporting of Observational Studies in Epidemiology (STROBE) guidelines for reporting observational (cross-sectional) research.

## References

[1] Al-Tawfiq J. (2025). Striving for zero traditional and non-traditional healthcare-associated infections (HAI): A target, vision, or philosophy. Antimicrob. Steward. Healthc. Epidemiol..

[2] Gidey K., Gidey M.T., Hailu B.Y., Gebreamlak Z.B., Niriayo Y.L. (2023). Clinical and economic burden of healthcare-associated infections: A prospective cohort study. PLoS ONE.

[3] Stewart S., Robertson C., Pan J., Kennedy S., Haahr L., Manoukian S., Mason H., Kavanagh K., Graves N., Dancer S.J. (2021). Impact of healthcare-associated infection on length of stay. J. Hosp. Infect..

[4] Zimlichman E., Henderson D., Tamir O., Franz C., Song P., Yamin C., Keohane C., Denham C., Bates D. (2013). Health care–associated infections: A meta-analysis of costs and financial impact on the US health care system. JAMA Intern. Med..

[5] Albarmawi M., Al Hadid L., Alnjadat R., Aljabery A. (2024). A multi-institute, follow-up, observational study measuring nursing students’ adherence to infection prevention and control protocols in Saudi Arabia. Front. Med..

[6] Yaas M.H., Hamarash M.Q., Almushhadany O.I., Ibrahim R.H., Jassim R.S. (2023). Assessing the effectiveness of clinical rotations in preparing undergraduate nursing students for practice: Mixed study. Malays. J. Nurs..

[7] Park E., Park H.-R., Lee J.-H. (2023). Barriers to learning healthcare-associated infections prevention and control during clinical practicum among nursing students in Korea: A focus group study. Int. J. Environ. Res. Public Health.

[8] Woo M.W.J., Cui J. (2025). Nursing Students’ Experiences and Perceived Learning Effectiveness of Patient Safety and Its Influencing Factors: An Integrative Literature Review. J. Adv. Nurs..

[9] Taylor D.C.M., Hamdy H. (2013). Adult learning theories: Implications for learning and teaching in medical education: AMEE Guide No. 83. Med. Teach..

[10] Ajzen I. (2011). The theory of planned behaviour: Reactions and reflections. Psychol. Health.

[11] Ward D.J. (2013). The application of the theory of planned behaviour to infection control research with nursing and midwifery students. J. Clin. Nurs..

[12] Hagger M.S., Cheung M.W.-L., Ajzen I., Hamilton K. (2022). Perceived behavioral control moderating effects in the theory of planned behavior: A meta-analysis. Health Psychol..

[13] Fishbein M., Ajzen I. (1977). Belief, Attitude, Intention, and Behavior: An Introduction to Theory and Research.

[14] Sin C.S., Rochelle T.L. (2022). Using the theory of planned behaviour to explain hand hygiene among nurses in Hong Kong during COVID-19. J. Hosp. Infect..

[15] Lee J., Kang S.J. (2020). Factors influencing nurses’ intention to care for patients with emerging infectious diseases: Application of the theory of planned behavior. Nurs. Health Sci..

[16] Kim N.Y., Jeong S.Y. (2021). Perioperative patient safety management activities: A modified theory of planned behavior. PLoS ONE.

[17] Pangaro L., Ten Cate O. (2013). Frameworks for learner assessment in medicine: AMEE Guide No. 78. Med. Teach..

[18] Pegram A., Bloomfield J. (2015). Infection prevention and control. Nurs. Stand..

[19] Liu L.-M., Curtis J., Crookes P.A. (2014). Identifying essential infection control competencies for newly graduated nurses: A three-phase study in Australia and Taiwan. J. Hosp. Infect..

[20] Riaz S., Afzal M., Ali A., Khan S. (2023). Evidence-Based Practices of Nurses Regarding Nosocomial Infection in ICU. A Descriptive study: Practices of Nurses Regarding Nosocomial Infection in ICU. Pakistan J. Health Sci..

[21] Ekakoro N., Nakayinga R., Kaddumukasa M., Mbatudde M. (2025). Knowledge and attitude of nosocomial infection prevention and control precautions among healthcare personnel at Kiruddu Referral Hospital in Kampala, Uganda. BMC Health Serv. Res..

[22] Fortunka K., Strzelecka A., Król G., Paprocka P., Mańkowska A., Lesiak A., Karpeta U., Okła S., Spałek J., Kaliniak S. (2024). Knowledge and training needs in nosocomial infection among hospital staff in the City of Kielce, Poland: A Cross-Sectional Study. J. Nurs. Manag..

[23] Ayele D.G., Baye Tezera Z., Demssie N.G., Woretaw A.W. (2022). Compliance with standard precautions and associated factors among undergraduate nursing students at governmental universities of Amhara region, Northwest Ethiopia. BMC Nurs..

[24] Bouchoucha S.L., Phillips N.M., Lucas J., Kilpatrick M., Hutchinson A. (2021). An investigation into nursing students’ application of infection prevention and control precautions. Nurse Educ. Today.

[25] Mohammedi S.B., Gillois P., Landelle C. (2025). Nursing students’ knowledge and effectiveness of teaching in infection prevention and control. BMC Nurs..

[26] Al Maskari T.S., Al Barwani S., Al Alawi S.S., Al Reesi H.K., Alshidi A.S., Al Maskari M.A. (2022). Using the Theory of Planned Behaviour to assess nursing and allied health students’ knowledge and intention to care for patients with COVID-19. J. Clin. Nurs..

[27] Tawalbeh L.I., Al-Rawajfah O.M., Habiballah L. (2019). The Effect of Infection Control Course on Nursing Students’ Knowledge of and Compliance with Universal Precautions: A Quasi-experimental Study. Dimens. Crit. Care Nurs..

[28] Majidipour P., Aryan A., Janatolmakan M., Khatony A. (2019). Knowledge and performance of nursing students of Kermanshah-Iran regarding the standards of nosocomial infections control: A cross-sectional study. BMC Res. Notes.

[29] Kumar B. (2012). Theory of Planned Behaviour Approach to Understand the Purchasing Behaviour for Environmentally Sustainable Products.

[30] Gusti A., Isyandi B., Bahri S., Afandi D. (2015). Faktor determinan intensi perilaku pengelolaan sampah berkelanjutan pada siswa sekolah dasar. J. Kesehat. Masy. Andalas.

[31] Alrubaiee G.G., Baharom A., Faisal I., Shahar H.K., Daud S.M., Basaleem H.O. (2021). Implementation of an educational module on nosocomial infection control measures: A randomised hospital-based trial. BMC Nurs..

[32] Ministry of Health of the Republic of Indonesia (2017). Guidelines for Infection Prevention and Control in Health Care Facilities.

[33] Kemenkes R.I. (2021). Technical Guidelines for Infection Prevention and Control in Primary Health Care Facilities. https://infeksiemerging.kemkes.go.id/document/buku-pedoman-teknis-ppi-di-fktp-tahun-2020/view.

[34] Livshiz-Riven I., Hurvitz N., Ziv-Baran T. (2022). Standard precaution knowledge and behavioral intentions among students in the healthcare field: A cross-sectional study. J. Nurs. Res..

[35] Rahiman F., Chikte U., Hughes G.D. (2018). Nursing students’ knowledge, attitude and practices of infection prevention and control guidelines at a tertiary institution in the Western Cape: A cross sectional study. Nurse Educ. Today.

[36] Kim J.-M., Lee S.-H. (2012). Nursing students’ performance related to nosocomial infection control: An analysis based on the theory of planned behavior. J. Korean Acad. Soc. Nurs. Educ..

[37] Salu S., Okyere J., Charles-Unadike V.O., Ananga M.K. (2023). Nurses’ knowledge on nosocomial infections preventive measures and its associated factors in Ghana: A cross-sectional study. BMC Health Serv. Res..

[38] Jamiran N.S., Ludin S.M. (2021). Knowledge, Attitude and Practice on Infection–Control Among IIUM Kuantan Nursing Students During Coronavirus 2019 Disease (COVID-19) Outbreak. Int. J. Care Sch..

[39] Althiyabi F.S., Khuded F.M., Alzaidi F.M., Alswat A.S.G., Alotaibi F.S.B., Alotaibi W.S.B., Alotaibi K.I.A., Alshehri F.A.H., Almutairi A.M.A., Alnathli J.A.A. (2024). Assessment of nursing knowledge and practice toward prevention of acquired infections in the emergency department of King Faisal Medical Complex in Taif. SAGE Open Med..

[40] Oni O., Orok E., Lawal Z., Ojo T., Oluwadare T., Bamitale T., Jaiyesimi B., Akinjisola A., Apara T. (2023). Knowledge and perception of nosocomial infections among patients in a Nigerian hospital. Sci. Rep..

[41] Harris J.K. (2021). Primer on binary logistic regression. Fam. Med. Community Health.

[42] Twisk J.W.R. (2025). The Analysis of a Dichotomous Outcome Variable BT—Basic Principles of Applied Medical Statistics: A Practical Guide.

[43] Zhong Y., Zhao H., Wang X., Ji J. (2022). Using the theory of planned behaviour to explain junior nurses’ and final-year student nurses’ intention to care for COVID-19 patients in China: A multisite cross-sectional study. J. Nurs. Manag..

[44] Davran E., Karaca A. (2021). Knowledge Level of Nursing Students on Nosocomial Infection. J. Clin. Pract. Res..

[45] Lin S.-C., Ni L.-F., Wang Y.-M., Lee S.H., Liao H.-C., Huang C.-Y., Tseng Y.-C. (2021). Prelicensure nursing students’ COVID-19 attitude impact on nursing career decision during pandemic threat in Taiwan: A cross-sectional study. Int. J. Environ. Res. Public Health.

[46] Anuar T.N.A.T., Samsudin N., Rasudin N.S., Zain N.M. (2021). Knowledge and compliance regarding standard precautions among Nursing Students at Universiti Sains Malaysia. Int. J. Care Sch..

[47] De Almeida Lima A.B., Gusmão C.M.P., Lima L.D.E.S., De Macêdo Rocha D., Menegueti M.G., Silva A.D.O.E., Reis R.K. (2025). Effect of an educational intervention on nursing students’ knowledge about COVID-19 and compliance with standard and transmission-based precautions: A quasi-experimental study. BMC Nurs..

[48] Bartoníčková D., Kohanová D., Cakirpaloglu S.D., Steven A., Žiaková K. (2025). Exploring Nursing Students’ Perspectives on Patient Safety Culture in Clinical Settings: A Mixed-Method Study. J. Clin. Nurs..

[49] Subramaniam C., Johari J., Mashi M.S., Mohamad R. (2023). The influence of safety leadership on nurses’ safety behavior: The mediating role of safety knowledge and motivation. J. Saf. Res..

[50] Gagnon H., Hearn K., Tsang C., Yip E., Stuber L., Ile E., Bridger L., Saulnier G., Hanson H.M., Leal J. (2024). “We could have used a lot more of this before…”: A qualitative study understanding barriers and facilitators to implementing a provincial PPE safety coach program during the COVID-19 pandemic. Am. J. Infect. Control.

[51] Harrod M., Weston L.E., Gregory L., Petersen L., Mayer J., Drews F.A., Krein S.L. (2020). A qualitative study of factors affecting personal protective equipment use among health care personnel. Am. J. Infect. Control.

[52] Mark D., Khanna P. (2025). Perspectives on the use of personal protective equipment by acute care providers caring for patients on COVID-19 medical and critical care units. Can. J. Infect. Control..

[53] Gina N.S.V., Rasweswe M.M., Moagi M.M. (2024). Factors Affecting Student Nurses’ Adherence to Standard Precautions for Preventing Tuberculosis and HIV at Eswatini University. Open Public Health J..

[54] Cruz J.P. (2019). Infection prevention climate and its influence on nursing students’ compliance with standard precautions. J. Adv. Nurs..

[55] de Lourdes Lopes M., da Silva Lima T., da Silva Oliveira A.D., Amorim F.C.M., Sousa K.H.J.F., Figueiró R.F.S., Zeitoune R.C.G., Damasceno C.K.C.S. (2023). Nursing students’ knowledge and compliance with standard precautions. ACTA Paul. Enferm..

[56] Alshammari F., Cruz J.P., Alquwez N., Almazan J., Alsolami F., Tork H., Alabdulaziz H., Felemban E.M. (2018). Compliance with standard precautions during clinical training of nursing students in Saudi Arabia: A multi-university study. J. Infect. Dev. Ctries..

[57] Yeom G., Park J. (2024). Effectiveness of donning and doffing personal protective equipment education using video debriefing among Korean undergraduate nursing students. BMC Nurs..

[58] Abdulaziz A.A., Ibrahim M., Nemer F., Ghallab Z.A., Alsulami A.A., Alghamdi A.A., Samm H.A., Alasmari N.S., Alshamrani A.A., Aljohani S.H.S. (2025). Personal protective equipment usage and compliance among healthcare workers. Int. J. Community Med. Public Health.

[59] Yuan Y., Tang H., Ni F., Xu X., Peng Y. (2025). Mediating role of professional identity between clinical learning environment and patient safety attitudes and professionalism in nursing interns. Nurse Educ. Today.

[60] Khoshakhlagh A.H., Malakoutikhah M., Park J., Kodnoueieh M.D., Boroujeni Z.R., Bahrami M., Ramezani F. (2024). Assessing personal protective equipment usage and its correlation with knowledge, attitudes, performance, and safety culture among workers in small and medium-sized enterprises. BMC Public Health.

