# Supplementary material for: Influence of Perceived Behavioural Control and Knowledge on Nursing Students’ Intention to Prevent Nosocomial Infections: A Cross-Sectional Study
Source: Nurs Rep. 2026 Apr 13;16(4):130. doi: 10.3390/nursrep16040130 (PMC13118518; doi:10.3390/nursrep16040130)
Supplement: Supplementary file 1 [file nursrep-16-00130-s001.zip › nursrep-4161736-supplementary.pdf]

SUPPLEMENTARY FILES
ALL INSTRUMENTS

Table S1. All Instruments used

ENGLISH VERSION

1. INTENSION SCALE

To what extent are you willing to implement safe environmental practices (safety environment behavior) in preventing nosocomial infections? Please rate your response on a 5-point scale, where 1 indicates “strongly unwilling” and 5 indicates “strongly willing.”

1 – Strongly unwilling | 2 | 3 | 4 | 5 – Strongly willing.

No	Statement
1	I perform hand hygiene using soap/hand rub before contact with the patient.
2	I perform hand hygiene using soap/hand rub after contact with the patient.
3	I use personal protective equipment (PPE) when performing interventions according to standard operating procedures (SOP).
4	After venous catheter insertion, I immediately dispose of the IV catheter needle in a safety box.
5	I wear gloves when administering injections directly to the patient.
6	I disinfect the injection site from the center outward.
7	I use transparent dressing to cover the insertion site.
8	I ensure that the intravenous fluid is flowing properly according to procedure before leaving the patient.
9	I replace the IV dressing if it appears dirty or if there is fluid leakage.
10	I replace intravenous fluids without touching the sterile area.
11	I use sterile gloves when inserting a urinary catheter.
12	During urinary catheter insertion, I use sterile single-use anesthetic lubricant gel.
13	I apply aseptic techniques using an alcohol swab when performing direct or indirect injections.
14	I observe signs of swelling, redness, or excessive pain at the IV cannula insertion site.
15	I dispose of blood-contaminated gauze in an infectious waste bin (yellow plastic bag).
16	I wash my hands with soap and running water after performing procedures involving contamination or body fluids, after removing gloves.
17	I dispose of sharp waste in a safety box.
18	When coughing or sneezing, I cover my mouth and nose with a disposable tissue.
19	I change my uniform into clean clothing after leaving the hospital.
20	I immediately empty a full urine bag using a urinal container and dispose of it into the toilet.
21	I recap used needles after use.
22	After performing suction on a patient, I soak the suction catheter in sterile fluid for reuse.
23	I wear gloves every time I meet a patient.
24	I use the same gloves when measuring vital signs for multiple patients in the same room.
25	I place the urine bag on the bed or patient’s thigh when transferring the patient or making the bed.

2. **Perceived Behavioural Control (PBC) Scale**

The following statements represent factors identified by nursing professional students (Ners) that are perceived as either facilitators or barriers in implementing safe environmental practices (safety environment behavior) for the prevention of nosocomial infections/HAIs. Please indicate the extent to which you agree that each of the following statements makes it difficult to perform these practices. Use a 5-point Likert scale, where 1 indicates "strongly disagree" and 5 indicates "strongly agree."

1 – Strongly disagree | 2 | 3 | 4 | 5 – Strongly agree

No	Statement
	**Supporting Factors**
1	The availability of waste bins in every area of the hospital.
2	The availability of changing facilities (changing rooms) in each unit.
3	The availability of adequate facilities in each patient room, such as sinks and soap for hand hygiene.
4	The provision of guidance from the hospital Infection Prevention and Control (IPC) team and ward managers.
5	The presence of monitoring in waste segregation and the use of sharp instruments.
6	The availability of specific training on the management of nosocomial infections.
	**Inhibiting Factors**
7	Limited funding to improve infection control facilities.
8	Suboptimal supervision of infection prevention practices.
9	Lack of motivation or encouragement from the hospital.
10	Limited availability of personal protective equipment (PPE) for students.

3. KNOWLEDGE QUESTIONNAIRE ON NOSOCOMIAL INFECTION/HAI PREVENTION

Instruction:

Please select the one answer that you consider to be the most correct.

1. An infection that occurs in a patient during the course of treatment in a hospital or other healthcare facility, where no infection was present or incubating at the time of admission, and which may also appear after discharge, is defined as:
a. Nosocomial infection
b. Community-associated infections (CAP)
c. Hospital infections
d. Healthcare-associated infections (HAIs)

2. Measures taken to prevent the risk of contamination through body fluids, blood, secretions, or non-intact skin, whether in patients who are diagnosed, suspected of infection, or colonized, are referred to as:
a. Standard precautions
b. Transmission-based precautions
c. Universal precautions
d. Community precautions

3. One of the simplest standard precautions and a key pillar of infection prevention and control (IPC) to prevent transmission through blood and body fluids is:
a. Hand hygiene
b. Personal protective equipment (PPE)
c. Environmental hygiene
d. Equipment decontamination

4. Standard precautions are part of IPC programs in hospitals. Which of the following correctly represents components of standard precautions?
a. Hand hygiene, communication techniques, equipment decontamination, staff health, linen management
b. Hand hygiene, PPE, disposable equipment hygiene, cough etiquette, staff health, linen management
c. Hand hygiene, PPE, safe injection practices, staff health, environmental hygiene, equipment decontamination
d. Hand hygiene, PPE, aseptic technique, decontamination of patient equipment, linen management, waste management, safe injection and prevention of sharps injury, patient placement, environmental hygiene, cough etiquette

5. The correct statement regarding the use of PPE is:
a. PPE does not need to meet safety and protection standards for healthcare workers/patients
b. PPE should not be shared between healthcare workers/individuals
c. PPE is removed only after all procedures for all patients have been completed
d. Hand hygiene is not necessary when gloves are used

6. Precautions applied to prevent and break the chain of transmission via contact, droplets, and airborne routes are referred to as:
a. Standard precautions
b. Transmission-based precautions

c. Universal precautions
d. Community precautions

7. Environmental cleanliness in a work unit is reflected in the 5S principles (Sort, Set in order, Shine, Standardize, Sustain). The term "clean" refers to:
a. Equipment arranged neatly according to its function
b. Cleanliness of equipment and work areas
c. Absence of unused items in the room
d. A comfortable working environment with good air circulation

8. One of the facilities used as part of standard precautions for managing infectious fluid spills, especially blood, is the provision of a spill kit. Which of the following is NOT included in a spill kit?
a. Personal protective equipment (PPE)
b. Absorbent cloth/material
c. Sterile gauze
d. Disinfectant solution

9. Proper waste management protects healthcare workers from exposure to infectious waste. The appropriate container for disposing of sharp waste after patient procedures is:
a. Cardboard box
b. Waste bin with yellow plastic bag
c. Safety box
d. Used infusion bottle

10. The correct sequence of decontamination for critical instruments is:
a. Initial pre-cleaning – cleaning – high-level disinfection (HLD)
b. Disinfection – cleaning – sterilization
c. Sterilization – cleaning – HLD
d. Initial pre-cleaning – cleaning – sterilization

11. Proper linen management is essential to prevent exposure to infectious fluids. The correct practice for handling contaminated linen is:
a. Placing contaminated linen into a dirty linen trolley with a yellow plastic bag
b. Placing contaminated linen into a dirty linen trolley with a black plastic bag
c. Placing linen on the floor after removal from the patient's bed
d. Disposing contaminated linen into an infectious waste bin

12. The principle of safe injection is "one needle, one medication, one patient." The meaning of "one needle" is:
a. One needle for multiple patients
b. One needle for multiple medications
c. One needle for one medication and one patient
d. One needle for all types of medications for one patient

13. The sterile handling of medications, needles/syringes, vials, ampoules, and patient equipment—from storage, preparation, to administration—to maintain sterility is referred to as:
a. Septic technique
b. Aseptic technique

c. Disinfection technique
d. Sterile technique

14. The correct sequence of cough/sneeze etiquette is:
a. Hand hygiene – cover mouth with tissue – wear a mask
b. Cover mouth and nose with tissue – wear a mask – perform hand hygiene
c. Cover mouth and nose with tissue – dispose of tissue – perform hand hygiene – wear a mask
d. Wear a mask – dispose into waste – perform hand hygiene – cover mouth with tissue

15. Phlebitis is a local inflammation at the site of peripheral venous insertion and is a complication of intravenous therapy. Infection caused by peripheral IV line insertion is referred to as:
a. CLABSI
b. VAP
c. CAUTI
d. PLABSI

INDONESIA VERSION

1. INTENTION SCALE

Sebarapa besar **keinginan anda untuk menerapkan pelaksanaan** pengelolaan lingkungan yang aman (safety environment Behavior) dalam mencegah infeksi nosocomial. Harap menilai pada skala 5 poin dengan 1 menjadi sangat tidak berkeinginan dan 5 sangat berkeinginan. **1-Sangat tidak berkeinginan ⎮2--- ⎮3--- ⎮ 4--- ⎮5-Sangat berkeinginan**

No	Pernyataan
1	Saya membersihkan tangan dengan sabun/handrub sebelum kontak dengan pasien
2	Saya membersihkan tangan dengan sabun/handrub sesudah kontak pasien
3	Saya menggunakan alat pelindung diri saat melakukan intervensi sesuai SOP
4	Setelah melakukan insersi vena, jarum IV chateter langsung saya buang ke Safety Box
5	Saya menggunakan sarung tangan saat melakukan injeksi langsung pada pasien
6	Saya mendisfektan area yang akan ditusuk dari arah dalam ke luar
7	Saya menggunakan *transparan dressing* untuk menutup area penusukan
8	Saya memastikan cairan infus menetes dengan lancar sesuai prosedur sebelum meninggalkan pasien
9	Saya mengganti balutan infus jika tampak kotor atau adanya rembesan cairan pada balutan
10	Saya mengganti cairan infus dengan tidak menyentuh area steril
11	Saya menggunakan sarung tangan steril pada pemasangan kateter urine
12	Pada pemasanagan kateter urine saya menggunakan jeli pelicin-anestetik **steril "singl**e use".
13	Saya menerapkan tindakan aseptic menggunakan swab alkohol saat melakukan tindakan injeksi langsung/tidak langsung
14	Saya mengobservasi adanya pembengkakan, kemerahan atau rasa nyeri yang berlebihan pada area penusukan IV Canula
15	Saya membuang kassa yang terkontaminasi darah pasien ke tempat sampah infeksius (kantong pelastik kuning)
16	Saya melakukan cuci tangan pakai sabun dan air mengalir setelah melakukan tindakan yang terkontaminasi dan cairan tubuh setelah melepaskan sarung tangan
17	Saya membuang limbah benda tajam ke safety box
18	Saat batuk/bersin, saya menutup mulut dan hidung dengan tissue sekali pakai
19	Saya mengganti baju dinas dengan baju bersih lainnya setelah keluar dari rumah sakit
20	Saya segera mengosongkan urine bag yang penuh menggunakan penampung urinal yang selanjutnya dibuang ke toilet.
21	Saya menyarungkan kembali jarum suntik habis pakai
22	Setelah melakukan suction pada pasien, saya merendam kateter suction pada com steril untuk persiapan pemakaian berikutnya
23	Saya menggunakan sarung tangan setiap kali akan bertemu pasien
24	Saya menggunakan sarung tangan yang sama saat melakukan pengukuran tanda vital pada beberapa pasien dalam satu ruangan
25	Saya meletakkan kantung urine ditempat tidur/paha pasien saat memindahkan pasien/ merapikan tempat tidur

2. **Perceived Behavioural Control (PBC)**

Pernyataan berikut merupakan factor factor yang telah diindetifikasi oleh mahasiswa profesi ners yang dipersepsikan sebagai factor Pendukung dan Penghambat mahasiswa dalam pelaksanaan pengelolaan lingkungan yang aman (safety environment behavior) dalam mencegah infeksi nosocomial/HAIs.

Harap menunjukan sejauh mana anda setuju bahwa pernyataan berikut ini menjadi sulit untuk dilaksanakan. Silahkan menilai pada skala 5 poin dengan 1 menjadi sangat tidak setuju dan 5 menjadi setuju.

1-Sangat tidak setuju |2--- |3--- | 4--- |5-Sangat setuju.

No	Pernyataan
	**FAKTOR PENDUKUNG**
1	Adanya tempat sampah di setiap sudut rumah sakit.
2	Adanya fasilitas ganti baju disetiap ruangan (ruang ganti)
3	Adanya fasilitas yang memadai di setiap ruangan pasien seperti wastafel dan sabun untuk melakukan hand hygiene
4	Adanya pengarahan dari tim PPI rumah sakit dan kepala ruangan
5	Adanya monitoring dalam pemilahan sampah dan penggunaan benda tajam.
6	Adanya pembelajaran khusus tentang pengelolaan infeksi nosocomial.
	**FAKTOR PENGHAMBAT**
7	Terbatasnya dana dalam meningkatkan fasilitas pengontrolan infeksi.
8	Masih belum maksimalnya supervisi penerapan pencegahan infeksi
9	Minimalnya motivasi dari pihak rumah sakit
10	Terbatasnya peralatan APD yang dimiliki mahasiswa

3. **Knowledge Scale**

Kuesioner pengetahuan Mahasiswa tentang pencegahan infeksi nosocomial/HAIs

Instruksi : Pilihlah salah dari pernyataan berikut yang anda anggap paling benar

1. Infeksi yang terjadi pada pasien selama proses perawatan di rumah sakit atau fasilitas pelayanan Kesehatan lainnya, dimana tidak ada infeksi atau dalam masa inkubasi saat masuk rawat, serta dapat muncul setelah pulang rawat , adalah pengertian dari :
   a. Infeksi Nosokomial
   b. Community Associated Infections (CAP)
   c. Hospital Infections
   d. Healthcare Associated Infections (HAIs)

2. Upaya mencegah resiko kontaminsi melalui cairan tubuh, darah, secret, kulit yang tidak utuh, baik pada pasien yang didiagnosis, diduga terinfeksi atau kolonisasi , adalah pengertian dari :
   a. Kewaspadaan Standar
   b. Kewaspadaan Transmisi
   c. Kewaspadaan Umum
   d. Kewaspadaan Masyarakat

3. Salah satu kewaspadaan standar yang paling mudah dilakukan dan merupakan pilar PPI untuk mencegah transmisi darah, produk darah dan cairan tubuh adalah :
   a. Kebersihan Tangan
   b. Alat Pelindung Diri
   c. Kebersihan Lingkungan
   d. Dekontaminasi Alat

4. Kewaspadaan Standar merupakan salah satu program PPI di rumah sakit. Yang termasuk ke dalam Kewaspadaan Standar adalah :
   a. Kebersihan Tangan, Tekhnik Komunikasi , Dekontaminasi Alat, Kesehatan Petugas,Pengelolaan linen
   b. Kebersihan Tangan APD, Kebersihan Alat Habis Pakai, Etika Batuk, Kesehatan Petugas,Pengelolaan linen
   c. Kebersihan Tangan, APD, Penyuntikan yang Aman, Kesehatan Petugas, Kebersihan Lingkungan, Dekontaminasi Alat
   d. Kebersihan Tangan, APD, Tekhnik Aseptik, Dekontaminasi Alat Habis pakai pasien, Pengelolaan Linen,Pengelolaan limbah,Penyuntikan yang Aman & cegah cedera benda tajam, Penempatan pasien,Kebersihan Lingkungan,Etika Batuk.

5. Pernyataan yang benar tentang penggunaan APD adalah
   a. Semua APD tidak harus memenuhi standar keamanan, perlindungan dan keselamatan petugas/pasien
   b. Tidak berbagi APD yang sama antara dua petugas/individu
   c. Lepaskan APD setelah semua prosedur dari semua pasien telah selesai dilakukan
   d. Kebersihan tangan tidak diperlukan karena sudah menggunakan sarung tangan

6. Kewaspadaan yang diterapkan untuk mencegah dan memutus mata rantai penularan penyakit lewat kontak, droplet dan udara adalah pengertian dari :
   a. Kewaspadaan Standar
   b. Kewaspadaan Transmisi
   c. Kewaspadaan Umum
   d. Kewaspadaan Masyarakat

7. Kebersihan lingkungan suatu ruangan di unit kerja tergambar dalam 5 R yaitu Rapih, Resik, Rajin, Ringkas, Rawat. Yang dimaksud dengan Resik adalah
   a. Peralatan tersusun rapi sesuai dengan fungsinya
   b. Kebersihan peralatan dan daerah kerja
   c. Tidak adanya barang dalam ruangan yang tidak difungsikan
   d. Lingkungan kerja dengan udara yang nyaman

8. Salah satu Upaya sarana untuk kewaspadaan standar terhadap adanya tumpahan cairan infeksius terutama darah dalam ruangan pelayanan pasien adalah dengan penyediaan spillkit. **Dibawah ini yang bukan termasuk isi dari spillkit adalah** :
   a. Alat Pelindung Diri
   b. Kain penyerap/perca
   c. Kassa steril
   d. Cairan desinfektan

9. Pengelolaan limbah yang benar akan melindungi petugas dari pajanan limbah infeksius. Yang merupakan sarana untuk pembuangan limbah benda tajam setelah dilakukan Tindakan pasien di rumah sakit adalah :
   a. Kardus bekas
   b. Tempat sampah dengan kantong plastik kuning
   c. Safetybox
   d. Botol infus bekas
10. Tahapan dari proses dekontaminasi alat jenis kritikal adalah
    a. Pre Cleaning Awal – pembersihan – Desinfeksi Tingkat Tinggi (DTT)
    b. Desinfeksi – pembersihan – sterilisasi
    c. Sterilisasi – pembersihan - DTT
    d. Pre Cleaning Awal – pembersihan – sterilisasi

11. Pengelolaan linen rumah sakit harus benar untuk mencegah petugas terkontaminasi cairan infeksius dari linen yang bernoda. Prilaku yang tepat dalam penanganan linen kotor bernoda cairan infeksius rumah sakit adalah :
    a. Meletakkan linen kotor bernoda infeksius ke dalam trolly linen kotor warna kantong plastic kuning
    b. Meletakkan linen kotor bernoda infeksius ke dalam trolly linen kotor warna kantong plastic hitam
    c. Meletakkan linen kotor di lantai setelah dilepas dari Kasur tempat tidur pasien
    d. Membuang linen kotor bernoda infeksius ke dalam tempat sampah infeksius

12. Prinsip dari Penyuntikan yang Aman one needle, one medications, one patient. Yang dimaksud dengan one needle adalah :
    a. Satu jarum untuk beberapa pasien
    b. Satu jarum untuk beberapa obat
    c. Satu jarum untuk satu jenis obat dan satu pasien
    d. Satu jarum untuk semua jenis obat dan satu pasien

13. Penanganan secara steril terhadap obat, jarum/spuit, vial, ampul, alkes pasien, mulai dari penyimpanan, persiapan sampai penyuntikan sehingga kesterilan tetap terjamin disebut dengan :
    a. Tekhnik Septik
    b. Tekhnik Aseptik
    c. Teknik desinfeksi
    d. Tekhnik Steril

14. Tahapan dalam melakukan etika batuk atau bersin adalah:
    a. Cuci tangan – tutup mulut dengan tissue- pakai masker
    b. Tutup mulut hidung dengan tissue – pakai masker – cuci tangan
    c. Tutup mulut hidung dengan tissue -buang tissue ke tempat sampah – cuci tangan – pakai masker
    d. Pakai masker – buang ke tempat sampah - cuci tangan – tutup mulut hidung dengan tissue

15. Plebitis merupakan inflamasi local pada daerah insersi vena perifer yang merupakan komplikasi terapi intravena. Infeksi yang disebabkan oleh pemasangan infus melalui vena perifer disebut :
    a. CLABSI
    b. VAP
    c. CAUTI
    d. PLABSI
